# Within‐season changes in habitat use of forest‐dwelling boreal bats

**DOI:** 10.1002/ece3.6253

**Published:** 2020-04-13

**Authors:** Ville Vasko, Anna S. Blomberg, Eero J. Vesterinen, Kati M. Suominen, Lasse Ruokolainen, Jon E. Brommer, Kai Norrdahl, Pekka Niemelä, Veronika N. Laine, Vesa Selonen, Thomas M. Lilley

**Affiliations:** ^1^ Finnish Museum of Natural History University of Helsinki Helsinki Finland; ^2^ Department of Biology University of Turku Turku Finland; ^3^ Ecology and Evolutionary Biology Faculty of Biological and Environmental Sciences University of Helsinki Helsinki Finland; ^4^ Biodiversity Unit University of Turku Turku Finland; ^5^ Department of Animal Ecology Netherlands Institute of Ecology (NIOO‐KNAW) Wageningen The Netherlands

**Keywords:** bats, boreal zone forests, habitat use

## Abstract

Bats utilize forests as roosting sites and feeding areas. However, it has not been documented how bats utilize these habitats in the boreal zone with methods afforded by recent technological advances. Forest structure and management practices can create a variety of three‐dimensional habitats for organisms capable of flight, such as bats. Here, we study the presence of boreal bats in a forest forming a mosaic of different age classes, dominant tree species, canopy cover, soil fertility, and other environmental variables, throughout their active season in the summer using passive ultrasound detectors. Our results indicate a preference for mature forest by *Eptesicus nilssonii* and a pooled set of *Myotis* bats*.* Both groups of bats also showed temporal changes in their habitat use regarding forest age. In June and July, both groups occurred more often in mature than young forests, but from August onwards, the difference in occurrence became less evident in *Myotis* and disappeared completely in *E. nilssonii*. In addition, *E. nilssonii* was more often present in forests with low canopy cover, and its occurrence shifted from coniferous forests to deciduous forests during the season. The results reflect the within‐season dynamics of bat communities and their ability to utilize different types of forest as environmental conditions change. Yet, the results most importantly emphasize the importance of mature forests to bat diversity and the need to conserve such environments in the boreal zone.

## Graphical abstract

Forests provide bats with food and shelter. Here, we investigate habitat selection in boreal bat species in forests. Although different bat species show variability in habitat selection, all bats appear to favor older forests.

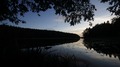

## 1 INTRODUCTION

The three‐dimensional arrangement of vegetation has an essential influence on habitat quality and, therefore, the presence and abundance of animal species at local scales (Tews et al., 2004). The complexity and diversity of vegetation determine the diversity and behavior of higher organisms by influencing the availability and diversity of resources and niches (Hekkala, Tarvainen, & Tolvanen, 2014), modifying microclimatic conditions (Melin, Matala, & Mehtatalo, 2014), or by providing sites for breeding and roosting (Fabianek, Simard, Racine, & Desrochers, 2015) and shelter or concealment from predators (Muiruri, Rainio, & Koricheva, 2016). The importance of forest structure on diversity has been recognized in the boreal zone, where industrial forestry has been an important economic driver for decades (Maanavilja, Aapala, Haapalehto, Kotiaho, & Tuittila, 2014; Noreika et al., 2015; Santangeli, Hogmander, & Laaksonen, 2013). Forest management practices determine the composition of forest ecosystems and the resources available for organisms inhabiting it. However, the use of these resources by cryptic animals in boreal forests, such as bats, has received less attention although they may use forest habitats in numerous ways and are an integral part of many forest ecosystems.

Forests are an important foraging habitat for a great diversity of bats (Luis Garcia‐Garcia & Santos‐Moreno, 2014; Melber, Fleischmann, & Kerth, 2013; Oporto, Arriaga‐Weiss, & Castro‐Luna, 2015), and almost half of the known bat species worldwide use trees as roosts for at least part of the year (Lacki, Hayes, & Kurta, 2007). Of European bats, almost all species utilize woodland for foraging or roosts, or both. Bats use trees as shelters, protection against predators, for social interactions (Kerth, Perony, & Schweitzer, 2011), as a likely means of reducing thermoregulation costs (Smith & Racey, 2005) and furthermore, they utilize the habitat trees create as a foraging area (Hillen & Veith, 2013). However, less is known about how the spatial arrangement of woodland patches or the composition of the woodland affects the presence of bat species, especially in the Palearctic boreal zone (Boughey, Lake, Haysom, & Dolman, 2011). Habitat complementation is a key process, determining the distribution of mobile species able to exploit non‐substitutable resources over large home ranges in heterogeneous landscapes. For instance, insectivorous bats need to forage in a diversity of habitat patches offering varied compositions and structures within forest landscape mosaics to fulfill their life cycle requirements (Charbonnier et al., 2015). In some cases, forest fragmentation can even benefit bat diversity by creating new foraging areas close to preferred roosting sites in cavities of trees and snags (Ethier & Fahrig, 2011; Mueller et al., 2013; Segers & Broders, 2014). Changes in landscape structure can therefore be expected to affect bat species diversity, abundance, and distribution (Law & Dickman 1998).

Bat diversity and abundance in forests are also affected by the age, diversity, and identity of the defining vegetation, which is dictated by forest management practices (Jung, Kaiser, Böhm, Nieschulze, & Kalko, 2012). Especially tree species identity as well as tree species diversity may enhance bat diversity through increased habitat heterogeneity and cascading effects on prey abundance and diversity (Tews et al., 2004). For instance, deciduous trees host a richer arthropod fauna compared to conifers (Mueller, Jarzabek‐Mueller, Bussler, & Gossner, 2014; Regnery, Couvet, Kubarek, Julien, & Kerbiriou, 2013). The accessibility of prey varies with tree density, height, and understory cover, with different effects expected among bat species and functional guilds (Jung et al., 2012; Mueller et al., 2013). However, coniferous forests dominate in the boreal zone and the tree species composition is different to studies conducted in the temperate zone. As food availability and roost density are two key resources for boreal bats, the species structure of tree stands and forests, governed by management practices, can be predicted to have a significant impact on the diversity of bat communities (Boughey et al., 2011; Russo, Cistrone, Garonna, & Jones, 2010). The age of the habitat forming vegetation, especially in various states of decay, also affects the diversity, abundance, and supply of insects (Gibb, Johansson, Stenbacka, & Hjalten, 2013).

The preference of many temperate bat species to forage in the vicinity of open waterbodies, lacustrine or riparian, has been previously documented (Bartonicka & Zukal, 2003; Ciechanowski, 2015; Gelhaus & Zahn, 2010). However, the significance of waterbodies may be weaker in the boreal zone, where forests, at least in their natural state, are often moist, sphagnum‐dominated bog‐like forests, often with seasonally exposed small waterbodies (Maanavilja et al., 2014; Maanavilja, Kangas, Mehtatalo, & Tuittila, 2015).Habitat use of bats in typical boreal forest environments such as spruce mires, dry pine‐dominated forests, or mixed deciduous woodlands has not been studied in detail earlier. Also, due to dramatic within‐season changes in lighting conditions, canopy cover, seasonal succession, and ground‐level humidity at high northern latitudes, there may be temporal differences in the use of habitats by bats depending on the resources available at a given time.

Here, we describe and discuss factors behind habitat use of bats in a boreal forest with forest patches differing in biotic and abiotic habitat characteristics. The study species present in the area are *Eptesicus nilssonii*, *Myotis brandtii*, *M. daubentonii,* and possibly *M. mystacinus*. However, because of the uncertainty involved in identifying *Myotis* bats to species level using acoustic data, we adopted to pool all *Myotis* and examine this group at the genus level. For instance, despite *M. daubentonii* being a trawling bat, which primarily feeds over water, it often roosts in abandoned woodpecker holes and may forage in forests while commuting to primary foraging sites (Dietz, Nill, & Helversen, 2009). The species may also forage in forests more during mid‐summer, when canopy cover offers them a higher degree of shade compared to open water.

We make several predictions on the habitat use of boreal bats based on prior knowledge outlined above and the temporal nature of our study. (a) We predict that in general, *E. nilssonii* favors more open habitats, such as seedtree stands. On the other hand, the *Myotis* will be detected more often in habitats with more canopy cover, particularly in mid‐summer, when the nights are very light. (b) We expect a temporal shift in the habitat use of the *Myotis* toward habitats with less canopy cover later during the season, as the length of the night, and therefore darkness, increases and these habitats become more suitable for the group. (c) We also expect higher number of *Myotis* observations in mature forests compared to young forests throughout the study season. Furthermore, (d) we expect a negative correlation between distance to waterbodies and the presence of bats, especially early in the season, as the abundance of insects is higher at permanent waterbodies. We expect this relationship to become weaker as overall insect abundance increases over the course of the season (Speakman & Rowland, 1999). Lastly, (e) we expect bats to more often utilize patches with deciduous trees and moist, productive soils, where the insect abundance is higher (Økland et al., 2005).

## 2 METHODS

### 2.1 Study site

**FIGURE 1:**
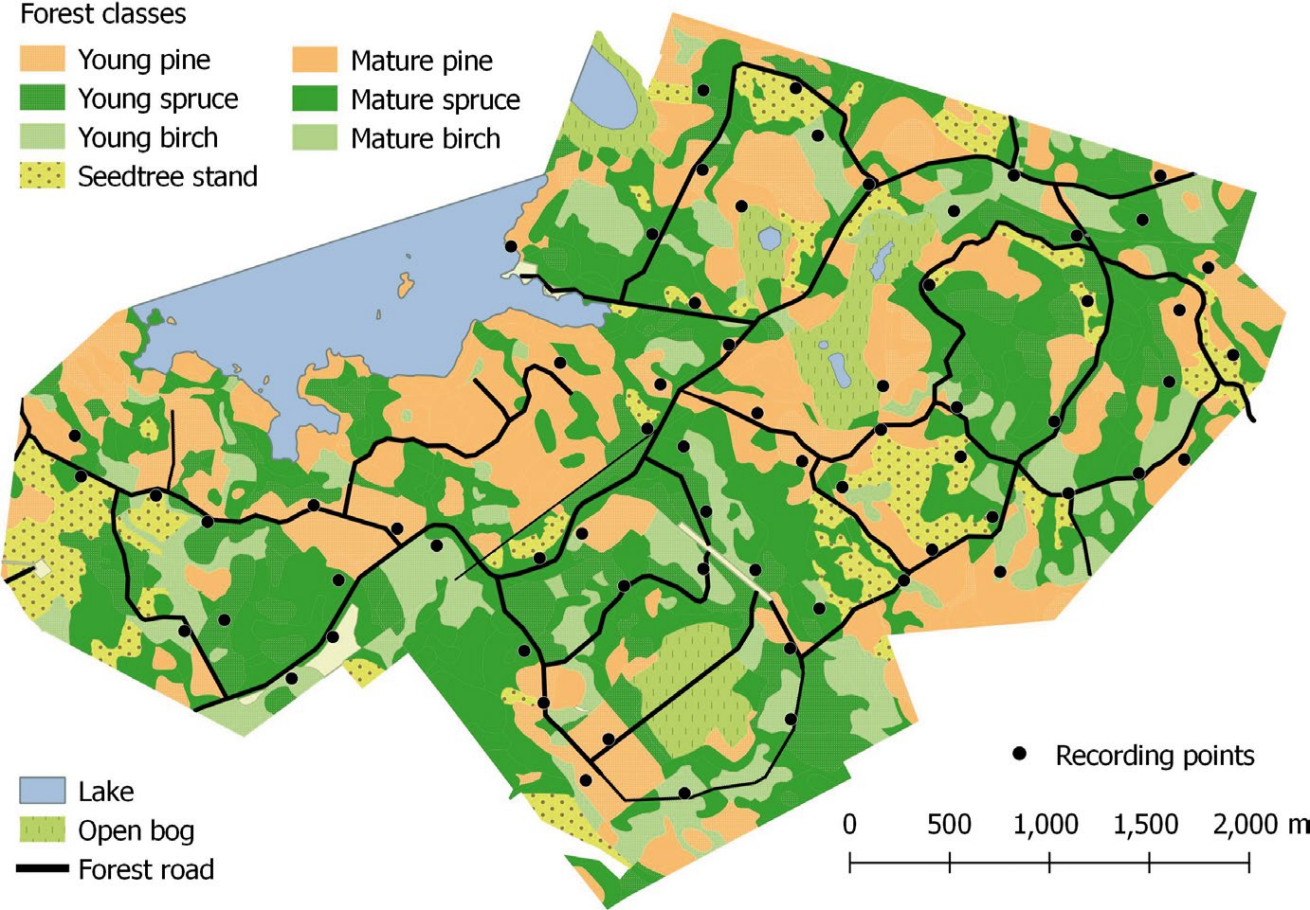
Map of the study area in Eurajoki, southwestern Finland, showing the land use and the location of the bat recorders in the area

The study was conducted in Eurajoki, southwestern Finland (N: 61°17′, E:21°45′), in the Metsähallitus (Parks & Wildlife Finland)—governed forest of Pinkjärvi (Figure 1) between 29 May and 3 October 2013. The 17 km^2^ forest area has been in fairly intensive commercial use until 2005 but is now protected. The area still resembles a managed forest to a large degree, consisting of three common tree species in boreal commercial forests: birch (*Betula pendula)*, pine (*Pinus Sylvestris*), and spruce (*Picea abies*). However, the area includes patches of mature forest of up to 140 years old. The study area was chosen for its range of forest patches of different age and dominant tree species, and its well‐documented history of management. Due to its previous commercial use, the forest is scattered with a network of small roads adding to the ease of equipment maintenance at the site. The area is neighbored by commercial forests in every direction excluding the northwest, where it is confined to a lake. Two smaller lakes surrounded by open bogs are included in the study area (Figure 1).

It is important to recognize that night length changes drastically during the summer months in northern latitudes. In our study area, the time from sunset to sunrise is on average only 4.5 hr in June and 5.5 hr in July, but already 8 hr in August and 11 hr in September (values from the 15th day of each month). During the shortest nights in June and July, even the darkest period of the night is lighter than the corresponding time point in August and September. The amount of light is one of the most important abiotic factors affecting the behavior of bats in our study area. Thus, month can also be considered as a proxy for increasing night length. In contrast, there was very little variation in the average nightly temperatures in our study area in 2013:12.1°C in June, 12.5°C in July, 11.6°C in August, and 7.7°C in September.

### 2.2 Study design

To study the temporal variation in forest habitat use by bats, we used GIS‐data provided by Parks & Wildlife Finland to coarsely divide the areas by age (<60 years old were labeled as “young” vs. >60 years old as “mature”) and dominant tree species (pine, spruce, birch). We selected 66 random locations equally divided into these six different habitats (Figure 1). Minimum distance between the locations was set to 200 m to prevent the detection of a single bat at multiple locations simultaneously. We used 33 passive acoustic recorders (Wildlife Acoustics SM2BAT+) to monitor bat activity at these locations from May to early October. In order to maximize the geographic area for data collection, and given the limited number of recorders, we decided to switch back and forth each recorded between two locations (A – B – A) once a week during which batteries and memory cards were also changed. This allowed to double the locations monitored (*n* = 66) given the number of available recorders (*n* = 33). The multidirectional ultrasound microphones were placed on tree trunks at a height of two meters. The recorders were set to begin monitoring an hour before sunset and end an hour after sunrise.

### 2.3 Bat identification

The season generated 21,358 recordings of bats in WAV format. The maximum length of one recording was set to 10 s. The data were then fed into Kaleidoscope (Wildlife Acoustics) software for automatically identifying bat echolocation calls up to species level. In the next phase, all identifications given by the program were manually verified and unidentified recordings were manually identified. Nights during which a species was not recorded at a given location were considered as absences. In 39 cases, it was not possible to determine the species in the recording, and nights containing only such recordings were treated as missing values. Due to challenges in separating detections between the different *Myotis* species, we grouped all detections of any *Myotis* species into a single group for a conservative approach. However, the identification of *Eptesicus nilssonii* was considered at single species level owing to the echolocation characteristics being very distinct from those of other Palearctic boreal bat species.

### 2.4 Environmental variables

The environmental variables used to predict bat presence were distance to closest waterbody, age of forest in 10‐year classes, canopy cover, and proportion of deciduous trees in the forest. Distance to waterbody was measured using GIS, with waterbody being defined as permanent standing water, excluding, for example, bogs, ephemeral ponds, and ditches. The age of the forest patches was obtained as a continuous variable from the database of the Parks & Wildlife Finland, where detailed information of every patch is updated regularly. In most cases, the exact age of the tree stand is available because the planting year has been recorded. The classification (young vs. mature) was not used in the final analyses. Canopy cover and proportion of deciduous trees were determined on site, at the scale of 300 m^2^ from the microphone. Canopy cover was estimated as the rough proportion of the sample area covered by tree canopy. The proportion of deciduous trees was obtained by counting the individual trees.

We assessed environmental conditions at the detectors with Ellenberg indicator values (EIV; Diekmann, 2003; Ellenberg et al., 1992) and the cover of bush and tree layers within a 10 m radius around the detectors. EIVs are based on the ranking of vascular plant species according to their optimum along the gradients for light, soil moisture, soil reactivity (reflecting soil calcium content), and soil productivity (Ellenberg et al., 1992). Plants have been given numerical values indicating their position along these ecological gradients; the indicator values for a site are calculated as a weighted average of all values of those species present in the plot (Diekmann, 2003). EIVs have been shown to be crude but reliable indicators, provided that the known pitfalls of the method are avoided (Diekmann, 2003). The ecological niches of understory plants tend to shift along latitudinal gradients (Wasof et al., 2013), and continentality may also play a role (Ellenberg et al., 1992). Therefore, we used EIVs adjusted for British conditions (Hill, Roy, Mountford, & Bunce, 2000) rather than the original values calculated for the central Europe (Ellenberg et al., 1992). In this study, we used EIVs for soil productivity and moisture, which we assumed to predict insect abundance.

### 2.5 Statistical analyses

To assess the habitat associations of the bat species, we ran two separate generalized linear mixed models (GLMM), one for all *Myotis* pooled (see above) and one for *Eptesicus nilssonii*. Models had a binomial distribution with logit link function. The GLMMs were ran using the function glmer in R package lme4. The sample unit for each analysis was the presence or absence for each recording night per species and per location. Sample size was 3,481 recording nights/location for *E. nilssonii* (1,096 presences and 2,385 absences) and 3,506 for *Myotis* (1,631 presences and 1,875 absences). Cases when the bat recorder had not been running the whole night (*n* = 10), due to rare technical issues, were excluded from the model. After this filtering, the average number of recording nights available per location was 53 nights for both species. In each model, week identity nested within location identity was included as a random factor to account for potential pseudoreplication of repeated observations within the same location in the same week. In each of the two GLMMs for each of the two species, we considered the same set of six continuous predictor variables: distance to the closest waterbody, age of the forest, canopy coverage, productivity (Ellenberg N), moisture (Ellenberg F), and coverage of deciduous trees. Prior to analyses, we scaled and centered all six predictors to zero mean and unit of variance. We also assessed the level of collinearity between the predictors by means of variance inflation factor (VIF) analyses for each of the two models separately. VIF values for all variables in each model were <1.5, indicating a very low level of collinearity (Zuur, Ieno, & Walker, 2009). Next, we built the full model (i.e., the one with the structure as above and including all main effects of the six predictors) and performed model selection and inference according to Burnham and Anderson (2003) using the package MuMIN (Barton, 2016) in R version 3.4.3 (R Core Team, 2015). Because model uncertainty was apparent after the above model selection (i.e., multiple models equally supported with ∆AIC < 2) for both GLMMs, we proceeded with multimodel averaging from across the subset of models with cumulative 95% AIC sum weight in both cases (Burnham & Anderson, 2003). Spatial autocorrelation of model residuals was assessed by visually inspecting spatial correlograms (Zuur et al., 2009), but no signs of spatial autocorrelation were apparent.

Once the overall habitat association between the two species of bats was established (see above), we then assessed whether these relationships varied between the four months (June to September) of the study being conducted. To achieve this, we ran a separate model for each species, including location identity as a random effect, because the month variable in the fixed part of the model already partly accounts for temporal patterns (see below). In each model with the above random structure, we included as predictor only the 2‐way interaction between month (with four classes) and in turn each of the six environmental predictors. As there were no missing values in the categorical (month) and all continuous variables, the number of locations per each month category was 66 across both species. The main effects of the variables forming the interaction were always included in each model.

## 3 RESULTS

**FIGURE 2:**
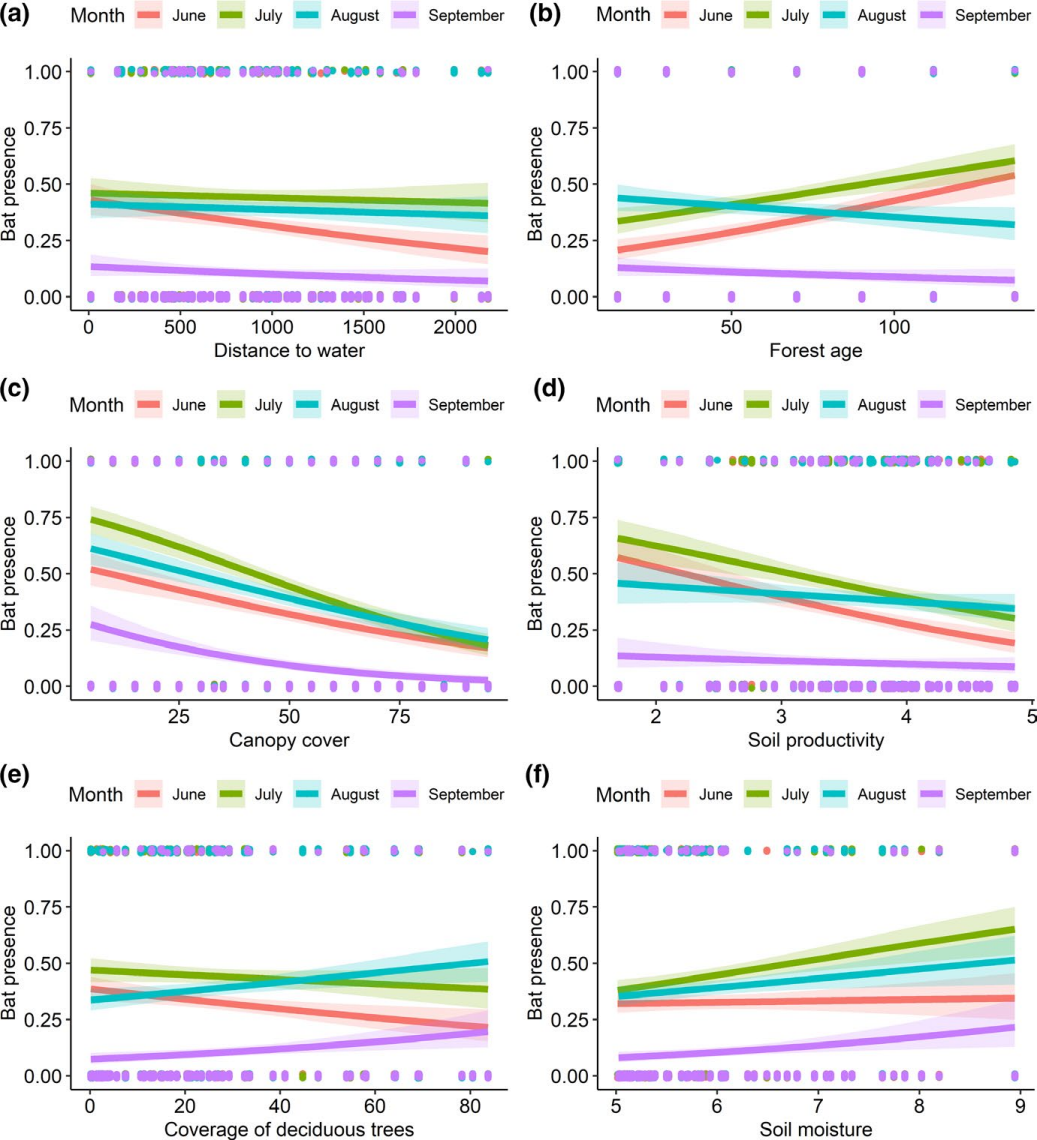
Association between predicted occurrence of *Eptesicus nilssonii* and each of the six environmental variables (a‐f) considered, presented separately for each month (lines of different colors). Continuous lines are derived from the predicted occurrence values resulting from each of the separate models including the interaction between month and each environmental predictor. The 95% confidence interval shading area is drawn based on the fixed part of each model. Dots represent the presence and absence points along the predictor variable range and are jittered vertically to ease visualization

**FIGURE 3:**
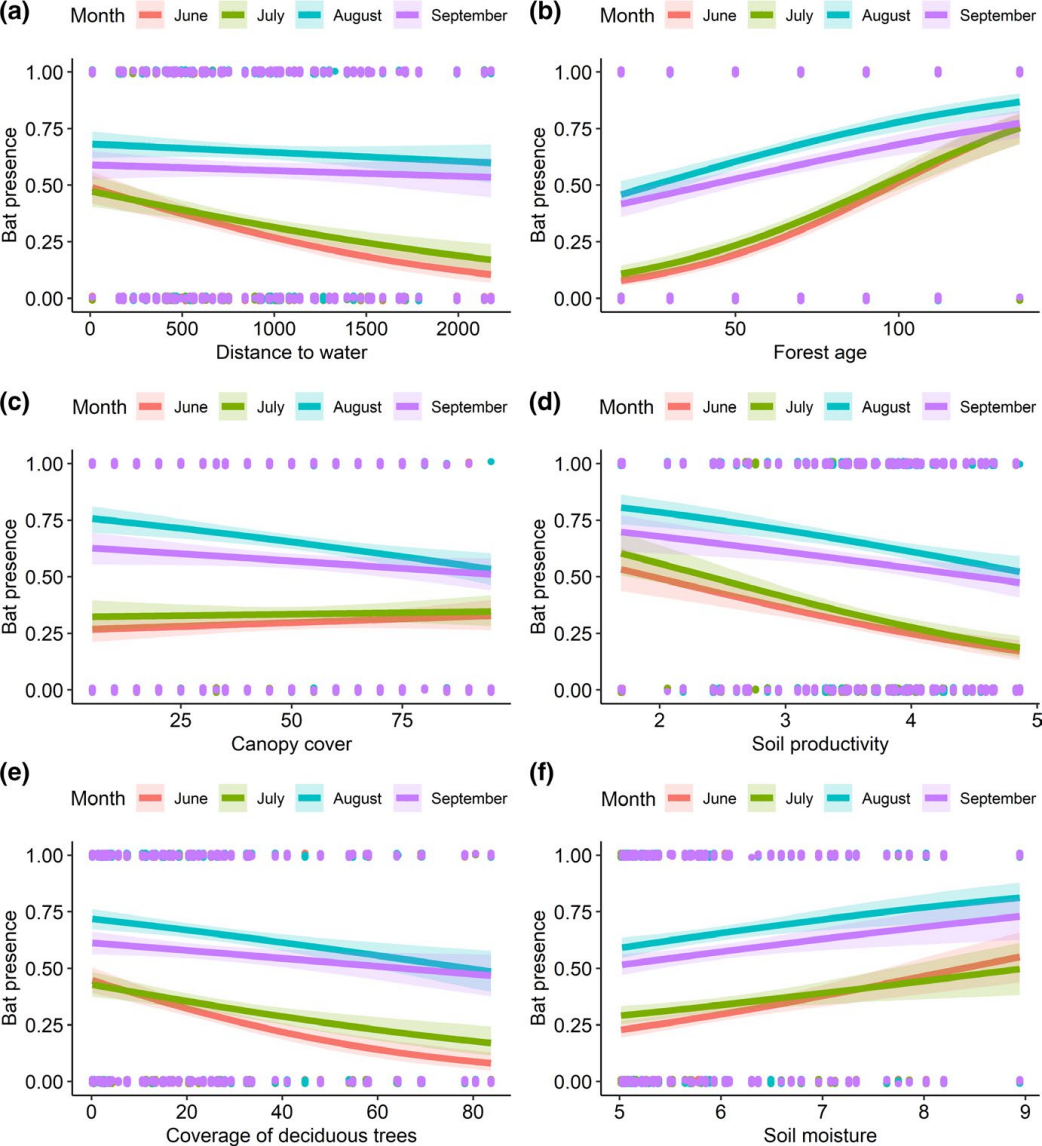
Association between predicted occurrence of *Myotis sp* and each of the six environmental variables (a‐f) considered, presented separately for each month (lines of different colors). Continuous lines are derived from the predicted occurrence values resulting from each of the separate models including the interaction between month and each environmental predictor. The 95% confidence interval shading area is drawn based on the fixed part of each model. Dots represent the presence and absence points along the predictor variable range and are jittered vertically to ease visualization

The occurrence of *E. nilssonii* in the study area was highest in July (Table 1), whereas for *Myotis,* the highest numbers were recorded in August (Table 2). As predicted, *E. nilssonii* were more often present in forests with less canopy cover (Table 3) and the grouped *Myotis* were more often present in mature forests (Table 5). Interactions between the months (Tables 4 and 6) showed that, in relation to the age of the forest, the behavior of both groups of bats changed during the season. In June and July, the occurrence of both groups of bats was higher in mature forests compared to young forests (Figures 2b and 3b). As the length of the night increased in August and September, the occurrence of *Myotis* increased in younger forests, but still remained higher in mature forests (Table 6, Figure 3b). For *E. nilssonii*, the seasonal change was more pronounced, as the effect of forest age on its presence disappeared after July (Table 4, Figure 2b). In addition, *E. nilssonii* was more often present in coniferous forests in June and July, but in August and September, the species was more often present in deciduous forests (Table 4, Figure 2e). However, contrary to our predictions, the *Myotis* did not occur in more open habitats as the season progressed. Neither soil moisture and soil productivity nor distance to water were important to either of the groups (Tables 3 and 5) and did not show any significant interactions with sampling month (Tables 4 and 6).

**TABLE 1:** Numbers of monthly *Eptesicus nilssonii* recording nights, observed presences, and average recordings per presence night according to the forest age classes

	*N* rec nights	*N* presences	Presence %	Average recs/presence	*SD* recs/presence
All					
June	872	286	32.8	14.0	41.9
July	835	368	44.1	13.1	40.8
August	895	348	38.9	10.6	30.7
September	879	94	10.7	1.4	1.0
Young					
June	391	93	23.8	3.2	3.3
July	360	131	36.4	5.3	8.5
August	389	157	40.4	4.1	7.1
September	384	46	12.0	1.2	0.5
Mature					
June	382	137	35.9	22.6	58.4
July	380	174	45.8	19.8	56.4
August	416	135	32.5	9.8	20.1
September	409	33	8.1	1.4	1.3
Seedtree					
June	99	56	56.6	10.7	15.8
July	95	63	66.3	10.6	23.2
August	90	56	62.2	30.7	65.4
September	86	15	17.4	1.7	1.2

**TABLE 2:** Numbers of monthly *Myotis* sp. recording nights, observed presences, and average recordings per presence night according to the forest age classes

	*N* rec nights	*N* presences	Presence %	Avg. recs/presence	*SD* recs/presence
All					
June	875	263	30.1	7.0	17.0
July	839	283	33.7	5.2	8.1
August	909	587	64.6	5.3	7.8
September	883	498	56.4	4.0	5.9
Young					
June	393	45	11.5	1.6	1.5
July	363	48	13.2	1.6	1.1
August	391	194	49.6	2.7	3.0
September	385	174	45.2	2.1	2.0
Mature					
June	383	192	50.1	8.7	19.6
July	381	201	52.8	6.7	9.2
August	427	330	77.3	7.1	9.6
September	412	281	68.2	5.6	7.3
Seedtree					
June	99	26	26.3	3.4	3.5
July	95	34	35.8	1.9	1.4
August	91	63	69.2	3.6	3.2
September	86	43	50.0	1.8	1.1

**TABLE 3:** The relationship between *Eptesicus nilssonii* occurrence and each of the five environmental variables considered as resulted from multimodel averaging (see methods for further details)

	Estimate	*SE*	*Z*	*p*
Intercept	−1.53	0.23	6.57	**<.001**
Distance to water	0.01	0.25	0.03	.975
Forest age	0.31	0.24	1.29	.197
Canopy cover	−1.05	0.24	4.47	**<.001**
Soil productivity	−0.26	0.25	1.03	.304
Deciduous trees	0.21	0.27	0.77	.441
Soil moisture	0.10	0.23	0.45	.648

High‐significance (*p* = < .001) indicated by bolded *p*‐values.

**TABLE 4:** The interaction between month (four classes, from June to September) and each of the six environmental variables, each tested in a separate model, in driving the occurrence of *E. nilssonii*

	LRT	*p*
Distance to water	5.27	.153
Forest age	48.16	**<.001**
Canopy cover	12.86	.005
Soil productivity	10.45	.015
Deciduous trees	48.48	**<.001**
Soil moisture	11.71	.008

Note

For the direction of the effects in each month, see Figure 2.

High‐significance (*p* = < .001) indicated by bolded *p*‐values.

**TABLE 5:** The relationship between *Myotis* occurrence and each of the five environmental variables considered as resulted from multimodel averaging (see methods for further details)

	Estimate	*SE*	*Z*	*p*
Intercept	−0.29	0.18	1.58	.114
Distance to water	−0.09	0.20	0.44	.661
Forest age	1.08	0.19	5.55	**<.001**
Canopy cover	−0.16	0.18	0.86	.391
Soil productivity	−0.20	0.20	1.00	.318
Deciduous trees	−0.04	0.21	0.21	.831
Soil moisture	0.26	0.18	1.44	.15

High‐significance (*p* = < .001) indicated by bolded *p*‐values.

**TABLE 6:** The interaction between month (four classes, from June to September) and each of the five environmental variables, each tested in a separate model, in driving the occurrence of *Myotis*

	LRT	*p*
Distance to water	13.64	.003
Forest age	33.83	**<.001**
Canopy cover	3.03	.387
Soil productivity	4.31	.23
Deciduous trees	15.32	.002
Soil moisture	2.81	.421

Note

For the direction of the effects in each month, see Figure 3.

High‐significance (*p* = < .001) indicated by bolded *p*‐values.

## 4 DISCUSSION

Our study provides the first in‐depth documentation of habitat use of bats in a boreal forest environment (although see Wermundsen & Siivonen, 2008). Despite the obvious shortcomings of not being able to reliably distinguish between the *Myotis* species present in the acoustic data, the results provide a clear distinction between habitat use between *E. nilssonii* and genus *Myotis*.

Differences between the study species in their preference of canopy cover can be attributed largely to their foraging behavior (Wermundsen & Siivonen, 2008). Whereas all species rely on aerial hawking for foraging over terrestrial environments (*M. daubentonii* trawls above waterbodies, Dietz et al., 2009), *E. nilssonii* prefers larger open spaces with less canopy cover (Dejong, 1994). However, the species is a generalist in its feeding behavior, which is represented by the plasticity of its echolocation call (Schnitzler, Moss, & Denzinger, 2003), allowing it to thrive in urban areas as well as rural and forest environments (Haupt, Menzler, & Schmidt, 2006; Wermundsen & Siivonen, 2008). The *Myotis* can be considered more as true forest species in the boreal zone (Wermundsen & Siivonen, 2008), which are considered to be adapted to a more cluttered environment with more canopy cover. One would expect canopy cover to be likely preferred because these species are deterred by light (Rydell, 1992; Stone, Jones, & Harris, 2009). This should be a problem especially for *Myotis* in northern latitudes during the summer, when the night is extremely short. For *Myotis*, avoidance of light has been experimentally demonstrated for bats flying along commuting routes (Stone, Jones, & Harris, 2012). The most widely accepted hypothesis explaining why these bat species avoid light is the fear of predators capable of hunting bats by visual cues since Myotis are considered to fly relatively slow (Stone, Harris, & Jones, 2015). Bats that do not need to forage early in the evening on crepuscular insect species tend to emerge later in the night when the light intensity has dropped further (Jones & Rydell, 1994; Rydell, Entwistle, & Racey, 1996). However, based on our results, we suggest *Myotis* did not appear to exploit the low light conditions dense canopy cover would have provided them, even during the short summer nights in the boreal zone. We speculate that the Myotis are not as sensitive to natural light as they are to artificial lighting and that forest, be it with less or more canopy cover, provides the species group with enough cover for foraging. In addition, the occurrences of Myotis in the patches with less canopy can also include individuals in transit flight, in which they probably cross areas not suitable for foraging.

The use of more mature forest is pronounced in the early season for both focal groups of bats. This is rather surprising considering *E. nilssonii*, which is very flexible in its habitat use outside forests, where it is found in a variety of urban and rural habitats (Dejong, 1994; Haupt et al., 2006). For *Myotis,* this was more expected, as more mature forest provides them with more protecting canopy, more day roost possibilities (Russo et al., 2010), and possibly even higher insect biomass and diversity (Martikainen, Siitonen, Punttila, Kaila, & Rauh, 2000). Indeed, we found that the *Myotis* prefer mature forest even in the autumn, which was not the case for *E. nilssonii*. Thus, mature forests appear to be relatively more important for the *Myotis*.

The use of more mature forest in both groups during early season could be explained by many factors. All species use forest clearings for feeding, only at different scales: The *Myotis* forage closer to the ground in small clearings created by individual trees dying and falling to the ground, or sometimes bigger groups of trees taken down by storms, whereas *E. nilssonii* typically flies higher, at canopy height, and exploits larger clearings or forages among tree tops. Clearings in all sizes are more common in more mature forests due to natural dynamics, and heterogeneity of the canopy structure is greater, thus offering more suitable foraging sites for both foraging groups. As a whole, mature forest appears to provide suitable foraging habitats for both groups of bats, with tall, old trees providing canopy height and flight space at the foraging height of *E. nilssonii*, as well as enough canopy cover to provide darkness and clutter at the foraging height of *Myotis*. These results suggest that forest managements, for instance, the age at which a forest is cleared, have a marked impact on the presence of bats in the boreal zone.

Earlier literature from the boreal zone associated the foraging of *E. nilssonii* with bodies of water (de Jong & Ahlén, 1991; Rydell, 1986). However, more recent studies have found the opposite (Ijäs, Kahilainen, Vasko, & Lilley, 2017; Wermundsen & Siivonen, 2008). Although these cases may be influenced by spatiotemporal and general environmental factors, the results from our study are more in line with the latter studies conducted in Finland with no significant association with distance to water observed. The *Myotis* also showed no association with distance to water, although a slight negative association can be observed from the figures for June and July, suggesting a preference of habitats close to waterbodies. This association is most likely heavily affected by the proportion of *M. daubentonii* in the acoustic data: a common bat species in southern Finland, which forages over water. Neither *M. brandtii* or *M. mystacinus* have been associated with proximity to waterbodies (Wermundsen & Siivonen, 2008), and in this respect, we can presume that only *M. daubentonii* contribute to the observed pattern. They most likely use the forests that are located close to waterbodies especially in June and July, when the areas of open water are not dark enough. The result could appear different if we were able to differentiate between the *Myotis* species.

Although the whole spectrum of soil productivity values (Ellenberg N) was available in the study area, we observed no effect of this on the presence of *E. nilssonii* and the *Myotis.* This was rather surprising, since productivity is expected to increase insect biomass. One possible explanation for this behavior could be the massive insect emergence in boreal forests during the summer months, offering abundant prey everywhere, even in the least productive sites. When prey abundance is not a limiting factor, the bats can select their feeding habitats based on other criteria, such as forest structure, vicinity to their roosts, and predator avoidance. Less productive sites could have properties which override the relatively lower insect biomass; for example, they most likely have less canopy cover (beneficial for *E. nilssonii*), but they might also contain more open space under the canopy, enabling *Myotis* to forage more effectively while still under the protection of trees.

Our results only apply to summer months and a rather restricted array of forested habitats. However, the results might imply that the *Myotis* indeed become more generalist in their habitat use toward autumn, as the nights become darker and they rely less on the protection provided by trees, allowing them to forage in less forested habitats. This is definitely known to be the case in *E. nilssonii*, which leave forested areas (and thus our study area) already during August to feed in more open habitats (Ijäs et al., 2017). Based on our results, similar behavior might occur in the *Myotis*, only taking place later, with reports of Myotis even far out on the Baltic Sea in August–September (Ahlen, Baagoe, & Bach, 2009).

Deciduous trees are able to host a richer arthropod fauna and more suitable microhabitats providing roost sites for bats than coniferous trees (Mueller et al., 2014; Regnery et al., 2013). If deciduous forests are available, they have been found to be preferred as foraging habitats over coniferous forests in Central Europe (Ciechanowski, 2015), and we also expected a similar result. Indeed, we found that *E. nilssonii* shifts between a preference from coniferous forests to deciduous forests in August and September. We observed no such trend for Myotis species, however, possibly due to our deciduous forests consisting of mainly of a single species, *Betula pendula*, which might not provide similar resources for Myotis as more diverse Central European forests (Ciechanowski, 2015). The scale of our study might also have been too detailed to observe this pattern; the areas dominated by deciduous trees might have to be larger in order to attract bats.

There may also be environmental variables which we were unable to measure, such as the effects of forest restoration in the area. These could partially explain some of the unexpected results. Another shortcoming of the study is that we only found a colony of *M. daubentonii* within the study area, but do not know where the closest breeding colonies of *M. brandtii* and *E. nilssonii* were located. Thus, it remains uncertain whether our results also apply to breeding females of these species, which might differ from nonbreeding individuals in their habitat requirements. However, radiotelemetry studies conducted on breeding *M. brandtii* females in Finland imply that they prefer similar habitats as in our study (P. Vihervaara, unpublished data).

The results accentuate the importance of mature forests in conserving biodiversity within a silvicultural context. Mature forests are important particularly for *Myotis* throughout the season. As a more generalist species, *E. nilssonii* prefers mature forests only during short summer nights. Bats are increasingly acknowledged as natural enemies that contribute significantly to the regulation of insect pests (Charbonnier, Barbaro, Theillout, & Jactel, 2014). Factors contributing to increased presence of bats in managed timber plantations can have a positive effect on yields.

## CONFLICT OF INTERESTS

No competing interests.

## AUTHOR CONTRIBUTION

**Ville Vasko**: Conceptualization (equal); Data curation (lead); Investigation (equal); Methodology (equal); Resources (equal); Writing‐original draft (equal); Writing‐review & editing (equal). **Anna Blomberg**: Conceptualization (supporting); Investigation (supporting); Resources (supporting); Writing‐review & editing (equal). **Eero J. Vesterinen**: Investigation (supporting); Methodology (supporting); Resources (supporting); Writing‐review & editing (supporting). **Kati Suominen**: Writing‐original draft (supporting); Writing‐review & editing (supporting). **Lasse Ruokolainen**: Conceptualization (supporting); Formal analysis (supporting); Resources (supporting); Writing‐review & editing (supporting). **Jon E. Brommer**: Formal analysis (supporting); Writing‐review & editing (supporting). **Kai Norrdahl**: Conceptualization (supporting); Data curation (equal); Investigation (equal); Methodology (equal); Writing‐original draft (supporting); Writing‐review & editing (supporting). **Pekka Niemelä**: Conceptualization (supporting); Funding acquisition (equal); Project administration (equal); Writing‐original draft (supporting); Writing‐review & editing (supporting). **Veronika N. Laine**: Conceptualization (supporting); Investigation (supporting); Writing‐original draft (supporting); Writing‐review & editing (supporting). **Andrea Santageli**: Conceptualization (supporting); Data curation (supporting); Formal analysis (lead); Methodology (supporting); Software (equal); Visualization (lead); Writing‐original draft (supporting); Writing‐review & editing (supporting). **Thomas M. Lilley**: Conceptualization (equal); Funding acquisition (equal); Investigation (equal); Methodology (equal); Project administration (lead); Resources (lead); Supervision (lead); Validation (lead); Writing‐original draft (equal); Writing‐review & editing (equal). **Vesa Selonen**: Conceptualization (supporting), Investigation (supporting), Resources (supporting), Writing ‐ review & editing (equal)

## Data Availability

The publication contains no data for public access.
